# Editorial: Extracellular vesicles as means of the activation, metabolic change induction, and remodeling of the tumor microenvironment

**DOI:** 10.3389/fonc.2024.1407949

**Published:** 2024-04-11

**Authors:** Michela Pozzobon, Alfredo Cappariello

**Affiliations:** ^1^ Department of Women’s and Children’s Health, University of Padua, Padua, Italy; ^2^ Foundation, Institute of Pediatric Research Città della Speranza, Padua, Italy; ^3^ Department of Life, Health and Environmental Sciences, University of L’Aquila, L’Aquila, Italy

**Keywords:** extracellular vesicles, tumor microenvironment, tumor niche remodeling, cancer progression, clinical outlooks

Extracellular vesicles (EVs) are cell-derived complex bi-phospholipidic structures incorporating active biomolecules derived from donor cells. The complex composition of EVs grants these molecules specific effects. Many studies identified specific roles of EVs arising from tumor cells in the crosstalk among cancer, resident cells and their extracellular matrix, contributing to the predisposition of the tumor microenvironment and finally favoring cancer aggressiveness and metastatization.

In the present Research Topic, we gathered articles highlighting the relevance of EVs in tumor spreading as well as their use as a diagnostic tool, arguing critically outlooks and limitations.

In particular, the review article from Sohal and Kasinski highlights the heterogeneity of EVs by describing novel subtypes, such as migrasomes, mitovesicles, and exophers, and evolving components, such as the surface protein corona. Furthermore, an overview of the role of EVs in different stages of cancer, such as cancer initiation, metabolic reprogramming, extracellular matrix remodeling, angiogenesis, immune modulation, therapy resistance, and metastasis, is provided. The article identifies gaps in the current knowledge of EV biology in cancer, *i.e.* EVs contribution to cancer initiation, early mutational landscape, and precancerous inflammation. The article concludes with perspectives on EV-based cancer therapeutics and challenges associated with bringing them to the clinic: long-term safety, heterogeneity, cargo loading mechanisms, and technical challenges associated with large-scale EV production. In a future scenario, engineered EVs can represent a new therapeutic perspective because they can be used as trojan: nano-shuttles with the ability to deliver where needed the therapeutic. Seeking the most efficient modification method able to keep intact the high bioavailability, biocompatibility and low or absent immunogenicity is still an intense effort for the scientific community.

Diao et al. reviewed the potential of tumor-derived extracellular vesicles (EVs) and their nucleic acids as diagnostic biomarkers for prostate cancer (PCa). They found that clinical body fluid samples, such as blood and urine can be promising sources of diagnostic EV RNA and DNA markers, such as miR-141, miR-375, PCA3, and TMPRSS2:ERG. The authors discuss the challenges and opportunities of EV DNA biomarkers, concluding that EV nucleic acids have unique advantages in tumor diagnosis, such as being stable, easy to detect, and reflecting tumor heterogeneity and dynamics as well as the fine-tuned detection of tumor-specific mutations and amplifications. However, they also point out the technical and clinical barriers that need to be overcome, such as standardizing EV isolation and purification methods, validation of EV biomarker performance, and translation of EV biomarkers into real clinical practice. As some miRNAs become diagnostic markers for some cancers, such as breast cancer, also EVs have the potential to become disease biomarkers, if supported by technical improvements. For instance, nowadays with digital PCR, that allows absolute quantification of low-level nucleic acid targets, detection of a very low number of target molecules inside the EVs is already possible, but a reliable discrimination of meaningful targets from unspecific or confounding molecules has to be set.

Takeda et al. conducted an original research study on the complex function of extracellular vesicles (EVs) in promoting bone metastases in renal cell carcinoma (RCC). To this aim, they established a bone metastatic RCC cell line (786-O BM) by *in vivo* selection and compared its EVs with those from the parental cell line (786-O luc). They found that 786-O BM EVs promoted angiogenesis and gap formation in the capillary endothelium of bone marrow in mice, both *in vitro* and *in vivo*. By proteomic analysis, they identified aminopeptidase N (APN) as a candidate protein in EVs enhancing angiogenesis. Accordingly, APN-knockdown in 786-O BM cells reduced angiogenesis and endothelial gap formation induced by their EVs. They also measured APN levels in EVs isolated from RCC patients with or without bone metastasis and found higher APN levels in EVs from bone metastatic patients. Finally, the authors injected 786-O luc cells into the left ventricle of mice that were pretreated with 786-O luc or 786-O BM EVs observing an increased bone metastasis in mice pretreated with 786-O BM EVs for 12 weeks compared to those pretreated with 786-O luc EVs or PBS. The authors concluded that EVs from bone metastatic RCC promote angiogenesis and vascular remodeling in the bone marrow before disseminated tumor cell colonization and that these histological changes are mediated by EV-APN. They suggested that EV-APN could be a potential biomarker and therapeutic target for bone metastatic RCC.

Nesi et al. showed that EVs from activated monocytes can spontaneously direct the system of the acquired immunity to give stimuli toward the resolution of the pathological situation. In particular, they presented a novel therapeutic approach for solid cancer treatment that exploits the capability of activated monocytes to transfer to cancer cells the MHC-I molecules bound to antigenic peptides, making the tumor a selected target of a natural CD8+ T cytotoxic lymphocytic response. The authors show that monocytes, either derived from cell lines or human donors, can transfer MHC-I molecules to cancer cells using microvesicles as cargo and that this transfer is enhanced by the activation of monocytes with tumor-conditioned media. The transferred MHC-I molecules can present exogenous antigens, such as ovalbumin or tetanus toxoid, to specific CD8+ T cells, which can then kill the cancer cells *in vitro* and *in vivo*. The proposed approach, based on explant and *in vitro* activation of immune cells from the same patients, could minimize biological, clinical and ethical controversies and concerns. Moreover, it can overcome some of the limitations of current immunotherapies, such as the lack or the relatively low immunogenicity of tumor-specific antigens, the systemic adverse effects, the immunosuppressive tumor microenvironment, and the high genetic drift of tumors. The useful dose of EVs is still an open question, being another major challenge for the therapeutic use of EVs in the future.

In this Research Topic we aim to present an overview of the potentiality, the stimulating advantages of the use of EVs as a theranostic platform in the cancer fight, as well as the still unsolved questions about their use as a concrete personalized medicine tool.

## Author contributions

MP: Conceptualization, Writing – review & editing. AC: Conceptualization, Data curation, Supervision, Writing – original draft, Writing – review & editing.

